# A simple analytical model for Neanderthal disappearance due to genetic dilution by recurrent small-scale immigrations of modern humans

**DOI:** 10.1038/s41598-025-22376-6

**Published:** 2025-11-04

**Authors:** Andrea Amadei, Giulia Lin, Simone Fattorini

**Affiliations:** 1 Department of Chemical Sciences and Technology, University of Rome Tor Vergata, Roma, 00133 Italy; 2 Department Aquatic Ecology, Eawag: Swiss Federal Institute of Aquatic Science and Technology, Dübendorf, 8600 Switzerland; 3 Department of Environmental Systems Science, ETH-Zürich, Zürich, 8092 Switzerland; 4 Department of Life, Health and Environmental Sciences, University of L’Aquila, L’Aquila, 67100 Italy

**Keywords:** Population genetics, Population genetics, Population dynamics

## Abstract

The disappearance of Neanderthals remains a subject of intense debate, with competing hypotheses attributing their demise to demographic decline, environmental change, competition with *Homo sapiens*, or genetic assimilation. Here, we present a mathematical model demonstrating that small-scale *Homo sapiens* immigrations into Neanderthal populations, providing recurrent gene mixing, could have led to almost complete genetic substitution over 10,000–30,000 years. Our model, grounded in neutral species drift, does not require selective advantage or catastrophic events but shows that sustained gene flow from a demographically larger species could account for Neanderthals’ genetic absorption into modern humans within a time-frame consistent with archaeological evidence. This scenario aligns with growing evidence of interbreeding and genetic introgression through recurrent *H. sapiens* immigration waves, providing a parsimonious explanation for the observed patterns of Neanderthal ancestry in present-day Eurasian populations. Although other factors may have contributed to the decline of Neanderthals, our results highlight genetic admixture as a possible key mechanism driving their disappearance.

## Introduction

The processes that led the modern humans to replace other hominins both culturally and genetically are under intense debate^1–3^, especially concerning the disappearance of Neanderthals (*Homo neanderthaliensis*) who originated in Eurasia around 400,000 years ago and lived in Europe and Western Asia as the predominant hominins until the arrival of *Homo sapiens* (modern humans)^4,5^.

Based on genetic and archaeological data, modern humans are thought to have migrated from Africa to the Levant (possibly earlier than 200,000 years ago^6,7^) and then spread throughout Eurasia by the end of the Middle Palaeolithic, around 45,000 years ago^8–11^. There, they came into contact with Neanderthals through subsequent immigration waves, possibly occurring over several thousand years^12,13^. The spread of modern humans has been suggested to be possibly linked to the disappearance of Neanderthals, probably occurred between 41,000 and 39,000 years ago^14,15^. However, reasons for Neanderthals’ extinction remain unclear^11^. Four primary groups of non-exclusive hypotheses have been proposed to explain the demise of the Neanderthals: (i) ‘demographic’, (ii) ‘environment-based’, (iii) ‘competition’ and (iv) ‘admixture’ hypotheses. The demographic hypotheses posit that Neanderthals’ extinction can be explained by demographic effects alone^5,16,17^. For example, Vaesen et al.^16^ suggested that the extinction of Neanderthals may have been driven by the small size and isolation of their populations, leading to inbreeding depression, reduced population growth rates, difficulties in finding mates (Allee effect) and stochastic fluctuations in birth rates, death rates and sex ratios. As a result, Neanderthal populations declined below the minimum viable population threshold even without the influence of modern humans. According to Degioanni et al.^17^, the extinction of Neanderthals might have been caused by a decrease in female fertility. Finally, Kolodny and Feldman^5^ proposed a model in which Neanderthals’ demise was determined by the population substitution due to the recurrent waves of *H. sapiens* expansion into Europe, eventually leaving no resources to the Neanderthal communities. All these hypotheses do not assume any role for the environment or inter-specific interactions, and are therefore sometimes indicated as ‘neutral’ or ‘null’ hypotheses, although it is impossible to fully separate demographic effects from those exerted by the environment or inter-specific interactions^3^. The environment-based hypotheses postulate that the Neanderthal meta-population may not have been able to survive large-scale climatic fluctuations across Europe and changes in vegetation patterns^2,18–20^, catastrophic climatic events^21^, or new diseases and pathogens introduced by *H. sapiens*^22,23^. The competition hypotheses focus on the competition between Neanderthals and modern humans for space and resources^5,24,25^, and how *H. sapiens* might have had advantages over Neanderthals due to better hunting strategies, broader diet or superior cognitive abilities and social structure^2,25–29^. Finally, the admixture hypotheses propose that Neanderthals and *H. sapiens* interbred producing fertile offsprings, eventually leading to Neanderthals being genetically absorbed into modern humans^4,30^.

The present modern human genome reveals multiple instances of genetic introgression with other hominins^31–34^, with growing evidence that interbreeding led to substantial gene flow between Neanderthals and modern humans^6^. Modern humans still retain a considerable amount of Neanderthal genetic material, at the species level if not in every individual^35^. *H. sapiens* major expansion in Eurasia occurred around 60,000–70,000 years ago, giving rise to all present-day non-African populations^36–38^. While earlier episodes of *H. sapiens* presence in Eurasia, in particular into the Levant over 100,000 years ago, are now believed to have involved lineages that ultimately became extinct, recent genomic studies have revealed that also early *H. sapiens* populations likely engaged in admixture with Neanderthals. However, there is no theoretical proof that genetic admixture alone can fully explain the loss of Neanderthals. Recent genetic analyses comparing present human genomes with the available Neanderthal ones have provided evidence of admixtures indicating *H. sapiens* $\rightarrow$ Neanderthals gene flows as early as 200,000-250,000 years ago^39–42^, also supporting the idea of recurrent immigrations of *H. sapiens* individuals into the Neanderthal settlements^6^. Such interbreeding events, although stemming from now-extinct *H. sapiens* lineages, may have initiated a long-term process of genetic exchange that was later amplified during the main out-of-Africa expansion.

Inspired by these recent results, we present a simple analytical model that explains the possible genetic dynamics underlying the replacement of Neanderthals by modern humans through successive *H. sapiens* immigration cycles into the Neanderthal communities, leading to Neanderthals’ genetic dilution. These immigration cycles (providing recursive genetic perturbations) are conceived as inward population fluxes from a virtually infinite *H. sapiens* demographic reservoir (in line with the classical continent-island model^43–48^), consistently with the modern human emigration from Africa over several thousand of years^5,6^. Although estimates of the total *H. sapiens* population in Eurasia during the Late Pleistocene vary considerably, ample evidence indicates that it was substantially larger than that of the Neanderthals. Some studies propose relatively small effective population sizes (typically ranging from 5,000 to 30,000 individuals) while others suggest much larger population sizes, from several tens of thousands to a few hundred thousand individuals, and in some cases even into the millions^2,49–51^. Therefore, the estimated size of the Neanderthal population of only a few thousands^3,6^, one or two orders of magnitude smaller than the *H. sapiens* population, justifies in our model the use of several modern human immigration cycles (providing recursive genetic perturbations) from an *infinite* *H. sapiens* demographic reservoir into the Neanderthal tribes (in practice, any modern human population much larger than the Neanderthal one would suffice for this model assumption to hold).

Our model avoids invoking disruptive events such as climate change or disease outbreaks, instead focusing on the impact of small-scale, recurring modern human immigrations into Eurasia, ultimately leading to the disappearance of Neanderthals’ genetic identity. The obtained deterministic mathematical framework, rooted in the analytical solutions for the mean population dynamics and focusing on the time-dependence of the corresponding genotype distribution, although sharing some basic premises, is different from classical continent-island models which describe discrete changes in allele frequencies (treated as a function of the generation number) within a single small population (the island) subject to immigration fluxes from an infinite population reservoir (the continent) and include stochastic terms to account for either genetic drift (as in Fisher-Wright models) or environmental variability (as in Gillespie’s extension), capturing the role of statistical noise in allele dynamics^44–48^. These models, typically requiring numerical solutions, provide the genetic variation of the single island population, rather than reconstructing the time evolution of the genetic distribution within an ensemble of independent populations (possibly exchanging individuals with their environment acting as a demographic/genetic bath) as in our approach. In fact, the ensemble probabilities of the genotypes of interest (i.e., the ensemble genetic distribution) that we obtain provide the genetic evolution of the Neanderthal population (ideally represented by the ensemble), as well as the genetic fluctuations of the single subpopulations in the ensemble due to statistical noise. Notably, our model assumes that the whole Neanderthal population was actually represented by a set of small interacting subpopulations, which is consistent with the general idea that Neanderthals were organized as a metapopulation^52,53^. Although estimates of the size of these groups are largely uncertain, there is ample evidence that they were characterized by low population densities^54–56^.

While acknowledging the genetic evidence of interbreeding between Neanderthals and modern humans, the model we present aims to demonstrate that small-scale genetic immigration events could be relevant to explain the observed patterns of Neanderthal ancestry in modern human populations. For the sake of simplicity, our model is based on the premise of neutral species drift, assuming no inherent selective advantage for either species.

## The mathematical model

### The demographic equation, its solutions and stationary conditions

The proposed model is entirely based on a number of basic definitions/approximations which can be summarized as follows.

- We consider a large (ideally infinite) set of equivalent, independent reference hunter-gatherer communities (reference tribes), each with identical initial population and embedded in a large environment allowing for immigration/emigration fluxes in/from the reference tribe, defining the statistical ensemble to be used.
- We define the expected demography of the tribe as the mean population dynamics obtained by averaging in the statistical ensemble (that is, averaging the population dynamics of the reference tribes).
- We always consider an exact female-male equality within the expected demography of the tribe.
- We assume that the tribe population essentially consists of two subpopulations: the non-reproductive subpopulation (i.e., children, elders, and infertile adults) and the reproductive subpopulation.

Therefore, defining with *N* the mean reproductive subpopulation per reference tribe (hereafter defined as the expected population with *N*/2 males and *N*/2 females) and assuming a simple mass action law model for the corresponding expected demography, we obtain the ordinary differential equation

1 $$\begin{aligned} \dot{N}= & k \; \frac{N}{2} \frac{N}{2} - k_E \; N - \gamma \; (N - N_{eq}) \nonumber \\= & \frac{k}{4} \; N^2 - k_E \; N - \gamma \; (N - N_{eq}), \end{aligned}$$

with $\dot{N} = d N/ d t$ the time derivative of the expected population, $\frac{k}{4} \; N^2$ accounting for the reproductive rate (that is, proportional to the number of possible reproductive female-male couples) and $- k_E \; N$ providing the exit rate including the death rate, the flux towards the older (non-reproductive) condition and the rate of adults becoming infertile for any reason. Furthermore, $- \gamma \; (N - N_{eq})$ corresponds to the migration rate (immigration and emigration) involving the stable equilibrium expected population $N_{eq} \ne 0$, with $k, k_E, \gamma \ge 0$ being the rate constants independent of *N*. It is worth to note that when considering $k_E$ and $\gamma$ as linearly dependent on *N*, then Eq. (1) can retrieve the familiar logistic growth equation^57^, as shown in the Appendix included in the Supplementary Information. In general such rate parameters can be modeled via Taylor expansions in *N* around $N_{eq}$, possibly including high order terms^58^. Given the lack of information on the dynamics of the Neanderthal populations and our focus on the demography of populations close to the stable equilibrium condition (i.e., $(N - N_{eq})/N_{eq} \approx 0$), we assume that only the expansion zero-th order term be relevant, thus considerably simplifying the derivations without loss of generality in the treatment of the genetic dynamics of a stationary expected population (see the next subsection).

$N_{eq}$, corresponding to the carrying capacity of the logistic growth equation, must provide a demographic equilibrium condition (that is, the stationary condition $\dot{N} = 0$) and thus from Eq. (1) we have

2 $$\begin{aligned} \frac{k}{4} \; N_{eq}^2 - k_E \; N_{eq} = 0, \end{aligned}$$

readily providing $N_{eq} = 4 k_E/k$. Using this last result when dividing Eq. 1 by $N_{eq}$ and $k_E$, we obtain

3 $$\begin{aligned} \frac{d f}{d s}= & f^2 - f - n (f - 1) = f^2 -(n+1) f + n \nonumber \\= & (f - 1) (f - n) \end{aligned}$$

4 $$\begin{aligned} f= & \frac{N}{N_{eq}} \end{aligned}$$

5 $$\begin{aligned} s= & k_E \; t = \frac{t}{\tau _E} \end{aligned}$$

6 $$\begin{aligned} n= & \frac{\gamma }{k_E}, \end{aligned}$$

where $f = 1$ (corresponding to the stable equilibrium expected population $N = N_{eq} = 4 k_E / k$) and $f = n$ (corresponding to the unstable equilibrium expected population $N = N' = 4 \gamma / k$) are the roots determining the stationary condition $d f/ d s = 0$. The dimensionless Eq. (3) can be solved yielding (for $n \ne 1$)

7 $$\begin{aligned} f(s)= & \frac{n (f_0 - 1) - (f_0 -n) \; e^{(n- 1) s}}{f_0 - 1 - (f_0 -n) \; e^{(n-1) s}} \end{aligned}$$

8 $$\begin{aligned} f_0= & f(0) = \frac{N(0)}{N_{eq}}. \end{aligned}$$

**Fig. 1:**
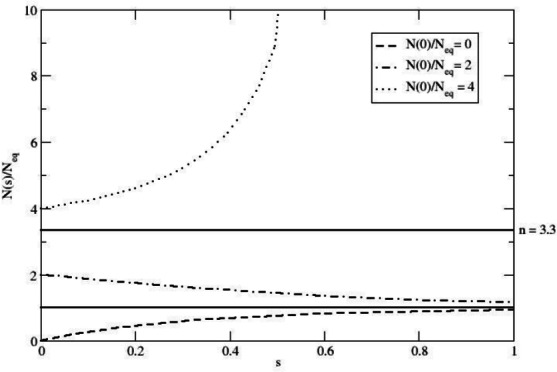
The function $f(s) = N(s)/N_{eq}$ (see Eq. 7) for different $f_0 = N(0)/N_{eq}$ values, using $n = \gamma /k_E = 3.3$. The Figure illustrates how the population size of a hypothetical Neanderthal expected tribe can change over the dimensionless *s* variable (i.e., the time as expressed in $\tau _E$ units). The different curves represent varying initial population sizes, demonstrating the system’s tendency to converge towards the stable equilibrium population when $0 \le f_0 \le n$, with the parameter *n* set to a possible reasonable value.

Once realizing that $n > 1$ (i.e., $\gamma > k_E$ ensuring that $N_{eq}$ provides the stable equilibrium condition), we find that *f*(*s*) always converges (as $s \rightarrow \infty$) to its stable equilibrium value $f = 1$ if $0 \le f_0 < n$ and diverges at

9 $$\begin{aligned} s = \frac{1}{n-1} \ln \frac{f_0-1}{f_0-n}, \end{aligned}$$

if $f_0 > n$, as illustrated in Fig. 1 (when $f_0 = n$ then *f*(*s*) is obviously stationary and thus fixed at the unstable equilibrium value $f = n$).

Hereafter, we will always consider $n > 1$ and $0 \le f_0 < n$, thus resulting in a virtually complete convergence of *f*(*s*) to $f = 1$ within a limited range of *s* (see Fig. 1), ensuring that the expected population can be treated as a fully stationary population with $N = N_{eq}$ (i.e., stable equilibrium condition).

### The genetic dynamics within a stationary population

Consider a stationary expected population with $N = N_{eq}$ and let us examine the distribution of genetic marker alleles which belong to either the Neanderthal or the *H. sapiens* species. Let us also consider that we can distinguish the corresponding amounts of Neanderthal homozygotes $N_N$, *H. sapiens* homozygotes $N_{S}$, and *H. sapiens*-Neanderthal heterozygotes $N_{NS}$ as functions of time and therefore $N_N(t) + N_S(t) + N_{NS}(t) = N_{eq}$. For simplicity and in line with Eq. 1 we make use of the following assumptions/approximations:

- We assume that for the chosen genetic marker, the three combinations of alleles (genetic subgroups) be characterized by the same reproductive *k* and exit $k_E$ rate constants, identical to those used for the entire expected population (i.e., the different alleles do not affect the fitness).
- We assume perfect male-female equality in each subgroup (i.e., the genetic marker under consideration does not belong to the sex chromosomes).
- We assume that the possible exchange of individuals between each reference tribe and its environment does not alter the distribution of the genetic marker alleles within the stationary expected population $N = N_{eq}$.

Therefore, introducing the probabilities of the three subgroups of the stationary expected population

10 $$\begin{aligned} P_N= & \frac{N_N}{N_{eq}} \end{aligned}$$

11 $$\begin{aligned} P_S= & \frac{N_S}{N_{eq}} \end{aligned}$$

12 $$\begin{aligned} P_{NS}= & \frac{N_{NS}}{N_{eq}}, \end{aligned}$$

with clearly

13 $$\begin{aligned} P_N + P_S +P_{NS}= & 1 \end{aligned}$$

14 $$\begin{aligned} \dot{P}_N + \dot{P}_S + \dot{P}_{NS}= & 0, \end{aligned}$$

and using for each genetic subgroup a rate equation similar to Eq. (1) with each pair of subgroups weighted by the appropriate Mendelian fraction within the reproductive term, we obtain (introducing the migration rate constants for the three genetic subgroups $\gamma _N = \gamma \; P_N, \; \gamma _S = \gamma \; P_S, \; \gamma _{NS} = \gamma \; P_{NS}$ such that $\gamma _N + \gamma _S + \gamma _{NS} = \gamma$)

15 $$\begin{aligned} \dot{P}_N= & \frac{\dot{N}_N}{N_{eq}} = N_{eq} \frac{k}{4} \Bigl \{ P_N^2 + P_N P_{NS} + \frac{1}{4} P_{NS}^2 \Bigr \} - k_E P_N \nonumber \\- & \gamma _N \Bigl ( P_N + P_S + P_{NS} - 1 \Bigr ) \nonumber \\= & k_E \Bigl \{P_N^2 + P_N \Bigl [1 - \Bigl (P_N + P_S\Bigr )\Bigr ] +\frac{1}{4} \Bigl [1 - \Bigl (P_N + P_S\Bigr ) \Bigr ]^2 - P_N \Bigr \} \nonumber \\= & k_E \Bigl \{\frac{1}{4} \Bigl [1 - \Bigl (P_N + P_S\Bigr ) \Bigr ]^2 - P_N P_S \Bigr \} \end{aligned}$$

16 $$\begin{aligned} \dot{P}_S= & \frac{\dot{N}_S}{N_{eq}} = N_{eq} \frac{k}{4} \Bigl \{ P_S^2 + P_S P_{NS} + \frac{1}{4} P_{NS}^2 \Bigr \} - k_E P_S \nonumber \\- & \gamma _S \Bigl ( P_N + P_S + P_{NS} - 1 \Bigr ) \nonumber \\= & k_E \Bigl \{P_S^2 +P_S \Bigl [1 - \Bigl (P_N + P_S\Bigr )\Bigr ] +\frac{1}{4} \Bigl [1 - \Bigl (P_N + P_S\Bigr ) \Bigr ]^2 - P_S \Bigr \} \nonumber \\= & k_E \Bigl \{\frac{1}{4} \Bigl [1 - \Bigl (P_N + P_S\Bigr ) \Bigr ]^2 - P_N P_S \Bigr \} \end{aligned}$$

17 $$\begin{aligned} \dot{P}_{NS}= & \frac{\dot{N}_{NS}}{N_{eq}} = N_{eq} \frac{k}{4} \Bigl \{\frac{1}{2} P_{NS}^2 + P_{NS} P_N + P_{NS} P_S + 2 P_S P_N \Bigr \} - k_E P_{NS} \nonumber \\- & \gamma _{NS} \Bigl ( P_N + P_S + P_{NS} - 1 \Bigr ) \nonumber \\= & - k_E \Bigl \{\frac{1}{2} \Bigl [1 - \Bigl (P_N + P_S\Bigr ) \Bigr ]^2 - 2 P_N P_S \Bigr \}\nonumber \\= & - \Bigl (\dot{P}_N + \dot{P}_S \Bigr ), \end{aligned}$$

where, due to the stable stationary condition, we used $N_{eq} = 4 k_E/k$ and

18 $$\begin{aligned} P_{NS} = 1 - \Bigl ( P_S + P_N \Bigr ). \end{aligned}$$

It is worth noting that even when assuming for $k_E$ and $\gamma$ any polynomial expression in $N-N_{eq}$, given the stationary condition Eqs. (15)-(17) would be correct.

From Eqs. (15) and (16) we readily obtain $\dot{P}_N = \dot{P}_S$ providing

19 $$\begin{aligned} P_N(t) = P_N(0) + P_S(t) - P_S(0), \end{aligned}$$

and thus

20 $$\begin{aligned} P_N(t) - P_S(t) = P_N(0) - P_S(0), \end{aligned}$$

clearly showing that $P_N - P_S$ is dynamically invariant. Therefore, introducing the function $Z(t) = P_N(t) + P_S(t) = P_N(0) - P_S(0) + 2 P_S(t)$ and using Eqs. (16) and (20) we obtain

21 $$\begin{aligned} \dot{Z}= & \dot{P}_N + \dot{P}_S = 2 \dot{P}_S \nonumber \\= & 2 k_E \Bigl \{\frac{1}{4} \Bigl [1 - \Bigl (P_N + P_S\Bigr ) \Bigr ]^2 - P_N P_S \Bigr \} \nonumber \\= & 2 k_E \Bigl \{\frac{1}{4} \Bigl [1 + \Bigl (P_N + P_S\Bigr )^2 - 2\Bigl (P_N + P_N\Bigr ) \Bigr ] - P_N P_S \Bigr \} \nonumber \\= & - k_E \Bigl (P_N + P_S \Bigr ) + \frac{k_E}{2} + \frac{k_E}{2} \Bigl [\Bigl (P_N + P_S\Bigr )^2 - 4 P_N P_S \Bigr ] \nonumber \\= & - k_E \Bigl (P_N + P_S \Bigr ) + \frac{k_E}{2} + \frac{k_E}{2} \Bigl (P_N - P_S\Bigr )^2 \nonumber \\= & -k_E \; Z + \frac{k_E}{2} \Big (\Delta P_0^2 + 1 \Bigr ) \end{aligned}$$

22 $$\begin{aligned} \Delta P_0= & P_N(0) - P_S(0), \end{aligned}$$

with solution

23 $$\begin{aligned} Z(t) = \Bigl [Z(0) - \frac{\Delta P_0^2 + 1}{2}\Bigr ] e^{-k_E t} + \frac{\Delta P_0^2 + 1}{2}, \end{aligned}$$

readily providing from

24 $$\begin{aligned} P_N(t)= & \frac{Z(t) + \Delta P_0}{2} \end{aligned}$$

25 $$\begin{aligned} P_S(t)= & \frac{Z(t) - \Delta P_0}{2} \end{aligned}$$

26 $$\begin{aligned} P_{NS}(t)= & 1 - Z(t), \end{aligned}$$

the time dependence of the subgroup probabilities

27 $$\begin{aligned} P_N(t)= & \Bigl [Z(0) - \frac{\Delta P_0^2 + 1}{2}\Bigr ] \frac{e^{-k_E t}}{2} + \frac{\Bigl (\Delta P_0 + 1\Bigr )^2}{4} \end{aligned}$$

28 $$\begin{aligned} P_S(t)= & \Bigl [Z(0) - \frac{\Delta P_0^2 + 1}{2}\Bigr ] \frac{e^{-k_E t}}{2} + \frac{\Bigl (\Delta P_0 - 1\Bigr )^2}{4} \end{aligned}$$

29 $$\begin{aligned} P_{NS}(t)= & - \Bigl [Z(0) - \frac{\Delta P_0^2 + 1}{2}\Bigr ] e^{-k_E t} + \frac{1 - \Delta P_0^2}{2}. \end{aligned}$$

**Fig. 2:**
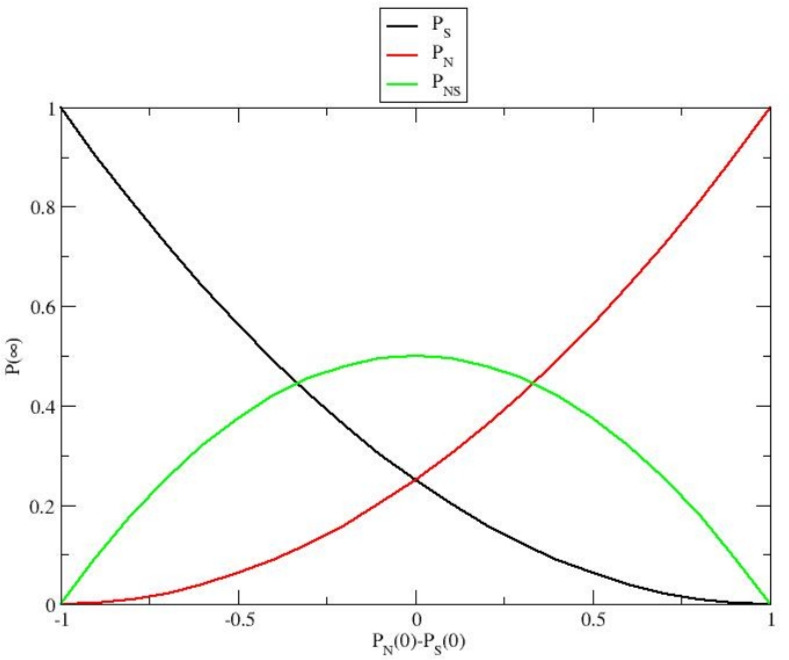
Long-term equilibrium probabilities of the different genetic subgroups within the expected population assuming interbreeding between Neanderthals and *Homo sapiens*. The *x*-axis represents the initial genetic composition, and the *y*-axis indicates the subgroup equilibrium probabilities (i.e., the fractions) $P_N(\infty )$ (Neanderthal homozygotes), $P_{S}(\infty )$ (*H. sapiens* homozygotes) and $P_{NS}(\infty )$ (the heterozygotes), see Eqs. (30)-(32).

From Eqs. (27)-(29) we then obtain the equilibrium genetic subgroup distribution within the stationary expected population by taking the $t \rightarrow \infty$ limit of the subgroup probabilities

30 $$\begin{aligned} P_N(\infty )= & \frac{\Bigl (\Delta P_0 + 1\Bigr )^2}{4} \end{aligned}$$

31 $$\begin{aligned} P_S(\infty )= & \frac{\Bigl (\Delta P_0 - 1\Bigr )^2}{4} \end{aligned}$$

32 $$\begin{aligned} P_{NS}(\infty )= & \frac{1 - \Delta P_0^2}{2} = \frac{-2 \Bigl (\Delta P_0 + 1\Bigr ) \Bigl (\Delta P_0 - 1\Bigr )}{4}, \end{aligned}$$

which are fully defined by $\Delta P_0$ (the initial difference between $P_N$ and $P_S$) and shown in Fig. 2. Note that the exponential relaxation of the subgroups within the stationary expected population is given by the mean lifetime $\tau _E = 1/k_E$, implying that, for any initial subgroup distribution, an approximate equilibrium condition is reasonably reached for $t > 2 \tau _E$.

### The effect of *Homo sapiens* immigration cycles on the equilibrium genetic subgroup distribution

The results described in the previous subsections clearly indicate that for reference tribes in ecological equilibrium with the environment (stable equilibrium condition), we can reasonably assume a stable genetic subgroup distribution within the stationary expected population, possibly modified only by input genetic perturbations altering the subgroup amounts and thus igniting a new genetic dynamics. In this paper, we always consider the tribe populations of the equilibrium ensemble to be initially made up of only the Neanderthal homozygotes (i.e., $N_N = N_{eq}$) with the immigration cycles due to *H. sapiens* homozygous individuals providing the input genetic perturbations within the stationary expected population (that is, the new *H. sapiens* homozygotes replace an equal number of individuals). In particular, if such immigration cycles occur with a mean time interval between two subsequent cycles larger than $2 \tau _E$, we can reasonably assume that each new cycle occurs when the genetic subgroup distribution has, at least approximately, reached the equilibrium condition after the previous cycle perturbation, thus allowing us to use the previous cycle equilibrium probabilities $P_N(\infty ), P_S(\infty ), P_{NS}(\infty )$ to obtain the new initial genetic condition. By assuming again that in each immigration cycle the new individuals within the expected population have female-male equality, we can express the initial genetic subgroup distribution in the *j*-th cycle (under the constraint of a stationary expected population $N = N_{eq}$) via

33 $$\begin{aligned} P_{S,j}(0)= & P_{S,j-1}(\infty ) + \frac{\delta }{N_{eq}} - P_{S,j-1}(\infty ) \frac{\delta }{N_{eq}} = P_{S,j-1}(\infty ) + \chi - P_{S,j-1}(\infty ) \chi \end{aligned}$$

34 $$\begin{aligned} P_{N,j}(0)= & P_{N,j-1}(\infty ) - P_{N,j-1}(\infty ) \frac{\delta }{N_{eq}} = P_{N,j-1}(\infty ) - P_{N,j-1}(\infty ) \chi , \end{aligned}$$

with $\delta$ the new *H. sapiens* homozygotes in each cycle and $\chi = \delta /N_{eq}$ (i.e., the *H. sapiens* homozygote immigration fraction). From the last equations, it follows (using also Eq. 20)

35 $$\begin{aligned} \Delta P_{0,j} = \Bigl [ P_{N,j-1}(\infty ) - P_{S,j-1}(\infty )\Bigr ] (1 - \chi ) - \chi = \Delta P_{0,j-1} (1 - \chi ) - \chi . \end{aligned}$$

From Eq. (35) we can then obtain by iteration (with $j \ge 1$)

36 $$\begin{aligned} \Delta P_{0,j}= & \Delta P_{0,0} (1 - \chi )^j - \chi \sum _{l = 0}^{j-1} (1 - \chi )^l = \Delta P_{0,0} (1 - \chi )^j - \Bigl [1 - (1-\chi )^j \Bigr ] \nonumber \\= & (1 - \chi )^j (1 + \Delta P_{0,0}) - 1, \end{aligned}$$

where we used the analytical expression of the geometrical sum

37 $$\begin{aligned} \sum _{l = 0}^{j-1} (1 - \chi )^l = \frac{1 - (1-\chi )^j}{\chi }, \end{aligned}$$

and clearly $\Delta P_{0,0}$ is the value of $\Delta P_0$ before any *H. sapiens* immigration cycle (that is, at $j = 0$) and thus $\Delta P_{0,0} = 1$.

**Fig. 3:**
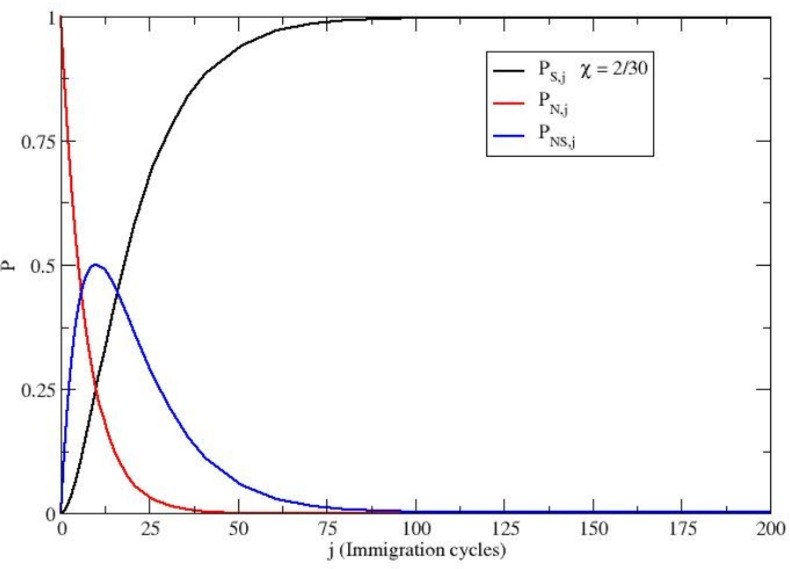
The equilibrium subgroup probabilities as a function of the *H. sapiens* immigration cycles (see Eqs. 38-40) using $\Delta P_{0,0} = 1$ and $\chi = 2/30$. The Figure shows how successive small-scale immigration events of *H. sapiens* progressively alter the equilibrium genetic composition of the expected population. Over multiple immigration cycles, the proportion of Neanderthal homozygotes sharply decreases, highlighting the process of genetic dilution.

From Eqs. (30) - (32) and employing Eq. (36) we have (with now $j \ge 0$ and setting $\Delta P_{0,0} = 1$)

38 $$\begin{aligned} P_{N,j}(\infty )= & \frac{\Bigl (\Delta P_{0,j} + 1\Bigr )^2}{4} = \frac{\Bigl [(1 - \chi )^j (1 + \Delta P_{0,0})\Bigr ]^2}{4} = (1 - \chi )^{2j} \end{aligned}$$

39 $$\begin{aligned} P_{S,j}(\infty )= & \frac{\Bigl (\Delta P_{0,j} - 1\Bigr )^2}{4} = \frac{\Bigl [(1 - \chi )^j (1+\Delta P_{0,0}) - 2\Bigr ]^2}{4} = \Bigl [(1 - \chi )^j - 1 \Bigr ]^2 \end{aligned}$$

40 $$\begin{aligned} P_{NS,j}(\infty )= & \frac{1 - \Delta P_{0,j}^2}{2} = \frac{1 - \Bigl [(1 - \chi )^j (1 + \Delta P_{0,0}) - 1\Bigr ]^2}{2} = \frac{1 - \Bigl [2 (1 - \chi )^j - 1\Bigr ]^2}{2}, \end{aligned}$$

providing the equilibrium probabilities of the genetic subgroups as a function of $\chi$ and the number of immigration cycles *j*.

It is useful to note that Eqs. (38)-(40) can be recast in terms of the allele frequencies carried by the ensemble to obtain the probabilities of sampling *H. sapiens* and *H. neanderthalensis* alleles after *j* immigration events.

It should be noted that Eqs. (38)-(40) furnishing the genotype distribution within the expected population can be used to obtain the corresponding probabilities $p_j$ and $q_j$ for the *H. sapiens* and Neanderthal alleles, respectively, after *j* immigration cycles

41 $$\begin{aligned} p_j= & \frac{2 P_{S,j}(\infty ) + P_{NS,j}(\infty )}{2[P_{S,j}(\infty ) + P_{NS,j}(\infty ) + P_{N,j}(\infty )]} = P_{S,j}(\infty ) + \frac{1}{2} P_{NS,j}(\infty ) = 1 - (1 - \chi )^{j} \end{aligned}$$

42 $$\begin{aligned} q_j= & \frac{2 P_{N,j}(\infty ) + P_{NS,j}(\infty )}{2[P_{S,j}(\infty ) + P_{NS,j}(\infty ) + P_{N,j}(\infty )]} = P_{N,j}(\infty ) + \frac{1}{2} P_{NS,j}(\infty ) = (1 - \chi )^{j}. \end{aligned}$$

The obtained expressions coincide with the allele-frequency recursion of the classical continent-island model under the assumption of a fixed modern human source (continent) population carrying the *H. sapiens* alleles to an island population initially fully characterized by the Neanderthal alleles, with a per-cycle immigration fraction $\chi$. Therefore, the evolution of allele frequencies provided by the continent-island model is recovered as a special result of our more general framework.

The results obtained by Eqs. (38)-(40) assuming $\chi = 2/30$ (we must consider only small genetic perturbations), are reported in Fig. 3. It is worth noting that $\chi$ corresponds in each immigration cycle to the average fraction of new *H. sapiens* homozygotes per reference tribe within the ensemble, thus possibly being even much smaller than $2/30 = 0.067$ as a result of the possible large number of reference tribes without new immigrant *H. sapiens* individuals at each immigration cycle.

When using Eq. (39) to obtain the total number of immigration cycles $J_f$ as a function of $\chi$ and the final probability of *H. sapiens* homozygotes $P_{S,f}$ (i.e., the probability of *H. sapiens* homozygotes $P_{S,j}(\infty )$ at the end of the immigration cycles), we obtain

43 $$\begin{aligned} J_f = \frac{\ln \Bigl (1 - \sqrt{P_{S,f}}\Bigr )}{\ln (1-\chi )}, \end{aligned}$$

**Fig. 4:**
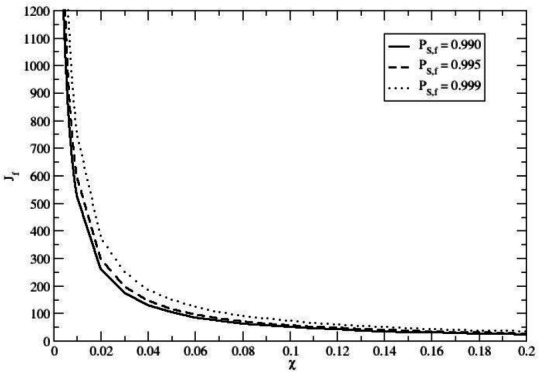
Total number of immigration cycles $J_f$ as a function of the fraction of *H. sapiens* newcomers per cycle $\chi$, required to achieve three different *H. sapiens* homozygote equilibrium probabilities within the final expected population (i.e., at the end of the immigration cycles): $P_{S,f} = 0.990, P_{S,f} = 0.995$ and $P_{S,f} = 0.999$. The results indicate that even a small, recurrent influx of *H. sapiens* homozygotes can drive the virtually complete disappearance of the Neanderthal allele.

**Fig. 5:**
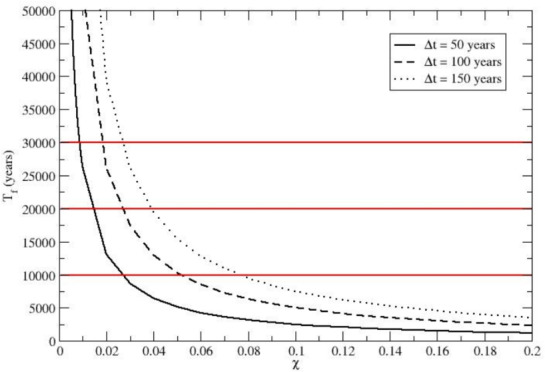
Estimates of total time $T_f$ needed for achieving the Neanderthal high genetic dilution (i.e., $P_{S,f} = 0.990$), as a function of $\chi$ (the *H. sapiens* immigration fraction per cycle). Results for three different mean time intervals $\Delta t$ between subsequent immigration cycles are shown. The Figure illustrates that over 10,000–30,000 years, recurrent small-scale *H. sapiens* immigrations could have led to the near-total demise of the Neanderthal alleles, thus resulting in a *H. sapiens* gene pool preserving only traces of the Neanderthal ancestry.

providing for each given $P_{S,f}$ the corresponding $J_f(\chi )$ function, as shown in Fig. 4 for $P_{S,f} = 0.990, P_{S,f} = 0.995$ and $P_{S,f} = 0.999$. Interestingly, for any value of $P_{S,f}$ (i.e., the final stationary fraction of *H. sapiens* homozygotes within the expected population) we can easily obtain the corresponding probability $P_{k_S}$ of finding $k_S$ reproductive individuals within a single reference tribe population of $N_{eq}$ reproductive individuals, corresponding to the *H. sapiens* homozygote subgroup

44 $$\begin{aligned} P_{k_S}= & \frac{N_{eq} ! }{k_S ! \; (N_{eq} - k_S)!} \; (P_{S,f})^{k_S} \; (1 - P_{S,f})^{N_{eq} - k_S} \end{aligned}$$

45 $$\begin{aligned} \langle k_S \rangle= & N_{eq} \; P_{S,f} \end{aligned}$$

46 $$\begin{aligned} \sigma _{k_S}^2= & N_{eq} \; P_{S,f} \; (1-P_{S,f}), \end{aligned}$$

readily providing

47 $$\begin{aligned} \sqrt{\langle \Bigl ( \frac{k_S}{N_{eq}} - \frac{\langle k_S \rangle }{N_{eq}} \Bigr )^2 \rangle } = \sqrt{\langle \Bigl ( \frac{k_S}{N_{eq}} - P_{S,f} \Bigr )^2 \rangle } = \frac{\sigma _{k_S}}{N_{eq}} = \frac{\sqrt{P_{S,f} \; (1-P_{S,f})}}{\sqrt{N_{eq}}}, \end{aligned}$$

and thus showing that for any reference tribe the statistical fluctuations of $k_S/N_{eq}$ decrease for increasing tribe population sizes, being negligible for $P_{S,f} \rightarrow 1$ (full *H. sapiens* homozygote population in each reference tribe). Such a statistical noise for the single reference tribe *H. sapiens* homozygote fraction within the final ensemble, can be conceived as stemming from the stochastic events acting on each reference tribe during the immigration cycles, thus mimicking the genetic drift effects. Not surprisingly, these statistical noise effects, providing deviations from the expected probability $P_{S,f}$ in a single reference tribe, tend to vanish as $P_{S,f} \rightarrow 1$ and/or $N_{eq} \rightarrow \infty$ (i.e., negligible genetic drift effects). Eq. (47) also readily provides the statistical noise (i.e., the standard deviation $\sigma _{\widehat{P}_{S,f}}$) for the estimate

48 $$\begin{aligned} \widehat{P}_{S,f} = \frac{1}{M} \sum _{l=1}^{M} \frac{k_{S,l}}{N_{eq}} = \Bigl ( \frac{1}{M} \sum _{l=1}^{M} k_{S,l} \Bigr )/N_{eq}, \end{aligned}$$

of the expected probability $P_{S,f}$ as obtained by averaging over a finite size sample of *M* reference tribes ($k_{S,l}$ being the number of *H. sapiens* homozygotes in the *l* reference tribe at the end of the immigration cycles)

49 $$\begin{aligned} \sigma _{\widehat{P}_{S,f}} = \sqrt{\langle \Bigl ( \widehat{P}_{S,f} - P_{S,f} \Bigr )^2 \rangle } = \frac{\sigma _{k_S}}{N_{eq} \sqrt{M}} = \frac{\sqrt{P_{S,f} \; (1-P_{S,f})}}{\sqrt{N_{eq} \; M}}, \end{aligned}$$

and hence, reasonably assuming a Gaussian-like statistics for $\widehat{P}_{S,f}$, we obtain within a 95 per cent confidence interval

50 $$\begin{aligned} P_{S,f} - 2 \frac{\sqrt{P_{S,f} \; (1-P_{S,f})}}{\sqrt{N_{eq} \; M}} \le \widehat{P}_{S,f} \le P_{S,f} + 2 \frac{\sqrt{P_{S,f} \; (1-P_{S,f})}}{\sqrt{N_{eq} \; M}}. \end{aligned}$$

The last equation furnishes the statistical noise (mimicking the genetic drift effects) for the final *H. sapiens* homozygote subgroup expected fraction as obtained by the more realistic finite size ensemble of reference tribes, and provides, for $P_{S,f} = 0.990$ with $\normalsize N_{eq} \approx 100, M \approx 10$ (likely the lower limit values of $N_{eq}$ and *M*), $0.984 \le P_{S,f} \le 0.996$ clearly showing that for a limited sample of reference tribes the genetic drift effects on the expected population (as obtained averaging over the reference tribes) are basically negligible when the immigration cycles granted a close to one $P_{S,f}$.

It is worth noting that the general expression for the probability $P(k_S,k_N,k_{NS})$ of finding $k_S$ *H. sapiens* homozygotes, $k_N$ Neanderthal homozygotes and $k_{NS}$ heterozygotes within a reference tribe population of $N_{eq}$ reproductive individuals is

51 $$\begin{aligned} P(k_S,k_N,k_{NS}) = \frac{N_{eq} ! }{k_S ! \; k_N ! \; k_{NS} !} \; (P_{S,f})^{k_S} \; (P_{N,f})^{k_N} \; (P_{NS,f})^{k_{NS}}, \end{aligned}$$

with obviously

52 $$\begin{aligned} P_{S,f} + P_{N,f} + P_{NS,f}= & 1 \end{aligned}$$

53 $$\begin{aligned} k_S + k_N + k_{NS}= & N_{eq}, \end{aligned}$$

providing a simple way to estimate the probability that in a single reference tribe of the ensemble the fractions $k_S/N_{eq}$, $k_N/N_{eq}$ and $k_{NS}/N_{eq}$ deviate from the corresponding expected probabilities $P_{S,f}, P_{N,f}, P_{NS,f}$ as a result of the random noise during the immigration cycles.

Finally, assuming a given mean time interval $\Delta t$ between two subsequent immigration cycles, we can obtain the total time $T_f(\chi )$ required to obtain the chosen $P_{S,f}$ (i.e., the final probability of *H. sapiens* homozygotes) via $T_f(\chi ) = J_f(\chi ) \Delta t$. In Fig. 5 we show such a $T_f(\chi )$ function for $P_{S,f} = 0.990$ using three different reasonable $\Delta t$ values: $\Delta t = 50, \Delta t = 100$ and $\Delta t = 150$ years. It is evident that to convert the average population of the reference tribe (the expected population) from an initially pure Neanderthal homozygote population to a 99 per cent *H. sapiens* homozygote population for a chosen genetic marker within time ranges of $10000-30000$ years, we need a *H. sapiens* homozygote immigration fraction $\chi$ within the range $0.008-0.08$, clearly showing that rather small recursive genetic perturbations could eventually have led to the disappearance of the Neanderthal alleles within an evolutionarily short time range (it must be remarked that for $P_{S,f} = 0.99$ the finite size ensemble noise is negligible for the expected population with all the reference tribes having a genetic subgroup distribution very close to the expected population one).

### The final distribution of *n* statistically independent genetic loci

In the previous subsections, we have derived the statistics for the *S*, *N*, *NS* subgroups of a given genetic marker (i.e., the allele combinations for a single genetic locus). Consider now a set of *n* statistically independent genetic loci (all falling within the conditions/approximations described earlier), each providing the three genetic subgroups. Given the same initial condition for all of them ($P_N = 1$), we see that the final probabilities $P_{S,f}, P_{N,f}, P_{NS,f}$ are identical for all genetic loci. Therefore, the probability $P_{n_S}$ that at the end of the immigration cycles a reproductive individual be *H. sapiens* homozygote for $n_S$ loci is

54 $$\begin{aligned} P_{n_S}= & \frac{n ! }{n_S ! \; (n - n_S)!} \; (P_{S,f})^{n_S} \; (1 - P_{S,f})^{n - n_S} \end{aligned}$$

55 $$\begin{aligned} \langle n_S \rangle= & n \; P_{S,f} \end{aligned}$$

56 $$\begin{aligned} \sigma _{n_S}^2= & n \; P_{S,f} \; (1-P_{S,f}), \end{aligned}$$

clearly showing that $\langle n_S \rangle \rightarrow n$ and $\sigma _{n_S} \rightarrow 0$ as $P_{S,f} \rightarrow 1$.

From Eq. (54) it follows that the probability for an individual to have at least one Neanderthal allele of the *n* genetic loci is given by $1 - (P_{S,f})^n$ and thus the probability $P_k$ of finding *k* reproductive individuals carrying in these loci Neanderthal alleles within a reference tribe population of $N_{eq}$ reproductive individuals is

57 $$\begin{aligned} P_k= & \frac{N_{eq} ! }{k ! \; (N_{eq} - k)!} \; [1 - (P_{S,f})^n]^k \; [(P_{S,f})^n]^{N_{eq} - k} \end{aligned}$$

58 $$\begin{aligned} \langle k \rangle= & N_{eq} \; [1 - (P_{S,f})^n] \end{aligned}$$

59 $$\begin{aligned} \sigma _{k}^2= & N_{eq} \; [1 - (P_{S,f})^n] \; (P_{S,f})^n, \end{aligned}$$

and hence

60 $$\begin{aligned} \frac{\langle k \rangle }{N_{eq}} = [1 - (P_{S,f})^n], \end{aligned}$$

with the corresponding standard deviation (i.e., the standard deviation of $k/N_{eq}$) given by

61 $$\begin{aligned} \sqrt{\langle \Bigl ( \frac{k}{N_{eq}} - \frac{\langle k \rangle }{N_{eq}} \Bigr )^2 \rangle } = \sqrt{\langle \Bigl ( \frac{k}{N_{eq}} - [1 - (P_{S,f})^n] \Bigr )^2 \rangle } = \frac{\sigma _{k}}{N_{eq}} = \frac{\sqrt{[1 - (P_{S,f})^n] \; (P_{S,f})^n}}{\sqrt{N_{eq}}}. \end{aligned}$$

Interestingly, while $\langle k \rangle /N_{eq}$ is always a decreasing function of *n* and $P_{S,f}$, $\sigma _{k}/N_{eq}$ has a maximum in *n* at $(P_{S,f})^n = 1/2$, being (as expected) a decreasing function of $N_{eq}$ and $P_{S,f}$. Such results readily provide for $P_{S,f} = 0.99, N_{eq} \approx 100$ and considering a limited number of independent loci (e.g., $n \approx 10$), $\langle k \rangle /N_{eq} \approx 0.1$ and $\sigma _{k}/N_{eq} \approx 0.03$. Therefore, within the expected population of a finite size sample of $M \approx 10$ reference tribes, we obtain for the ensemble mean of $k/N_{eq}$ (with expected value $[1 - (P_{S,f})^n]$) the standard deviation $\sigma _{k}/(N_{eq} \sqrt{M}) \approx 0.009$ clearly showing also for the case of *n* loci the negligible statistical noise within the expected population of a finite size ensemble.

## Discussion

The mathematical model we propose in this paper assumes that the metapopulation of Neanderthals was formed by basically independent tribes, each composed of several smaller and genetically interconnected communities (bands), occupying a fragmented habitat^3,52,59,60^. The 25-50 individual band size and the 500-1000 individual tribe size (i.e., 10-40 bands per tribe) are often considered as the typical population network for hunter-gatherers, possibly valid also for the Pleistocene human populations^5,61–63^, although the use of such an extrapolation to prehistoric human communities is controversial^61^. Bocquet-Appel and Degioanni^64^ proposed Neanderthal bands of about 50 individuals and a total Neanderthal population size of 5000-70,000 individuals. Kolodny and Feldman^5^ used a band/tribe size of 50-1000 individuals for both Neanderthals and *H. sapiens* with a total population of 5000-70,000 individuals (Neanderthals plus *H. sapiens*) in Europe for their demographic simulations. The reduced size of the Neanderthal population of only a few thousands^3,6^, one or two orders of magnitude smaller than the *H. sapiens* population, justifies in our model the use of recursive genetic perturbations affecting the Neanderthal tribes, due to several modern human immigration cycles.

We must remark that the analytical model employed is based on the ideal concept of the statistical ensemble (i.e., involving in principle an infinite statistical sample, see Subsection "The demographic equation, its solutions and stationary conditions"), allowing us to use the kinetic equations without considering any statistical noise (i.e., no genetic drift effects). The limited hunter-gatherer population size, especially for Neanderthals, makes such neglected noise possibly significant (i.e., significant genetic drift effects) for the single reference tribe and for the expected population dynamics when dealing with a finite size ensemble (i.e., a finite number of reference tribes). However, we showed that even for an ensemble of only 10 reference tribes, given a sufficient number of immigration cycles granting a close to one $P_{S,f}$ value, such a statistical noise is negligible for the ensemble mean population (the expected population) with all the reference tribes showing rather close genetic distributions.

We also acknowledge that the Neanderthal population structure was likely complex, with varying degrees of isolation across regions and time (e.g., late Western European Neanderthals had been isolated both from *H. sapiens* and other Neanderthal groups for tens of thousands of years^15^ and the Vindija Neanderthal ($\sim$ 40 kya) exhibits only $\sim$ 2 per cent *H. sapiens* ancestry, which is lower than what observed in the much older Altai Neanderthal ($\sim$ 120 kya)^6^. If the admixture mechanism we propose played a relevant role in Neanderthal demise, these data that appear at odds with our model could be explained by the fact that gene flow events did not occur uniformly across Neanderthal populations; cultural, ecological, or geographical factors may have created barriers to gene flow, leading to uneven introgression. Since our theoretical framework is not spatially explicit, such geographical variations are not explicitly treated, providing a *mean field* approximation of genetic dynamics within a representative statistical ensemble of Neanderthal tribes each embedded into a genetic/demographic bath. It is also worth noting that, in our model, the last period of Neanderthal presence ($\sim$ 40 kya) would have been characterized by an ensemble mean population already largely converted to the modern human genetic profile. Individuals that can still be genetically assigned to Neanderthals at this stage would therefore correspond to isolated communities with little or no modern human introgression, representing rare cases of tribes with a very unlikely genetic drift. A promising future direction would be to develop a spatially structured version of the model, capable of capturing local contact zones, migration barriers, and regional variations in admixture intensity.

There is no agreement on the duration of coexistence between the two species^5^, with reported coexistence periods as short as 5000-6000 years in Europe^3,5^. However, in general, a period of coexistence of at least 10,000 to 15,000 years is assumed in Europe^5^, with the first genetic admixtures in the Levant possibly occurring as early as 250 kya^6^ and the two species interacting for 20,000 years^3^ or more. Based on such data, we assumed that *H. neanderthaliensis* and *H. sapiens* potentially interbred at any location of co-occurrence through the entire period of coexistence, specifically considering the 10,000-30,000 year interval for the immigration cycles that provided the Neanderthal to *H. sapiens* genetic substitution. The notion that Neanderthals quickly disappeared following the expansion of *H. sapiens* into Eurasia^65^ has been challenged. A growing body of evidence suggests that their decline actually occurred at varying times across different regions^14^ over the entire period during which *H. sapiens* and *H. neanderthaliensis* coexisted^3^. Archaeological, anthropological and fossil evidence indicates that Neanderthal extinction was a gradual phenomenon, with the loss of local populations at different times. In general, it is assumed that *H. neanderthaliensis* disappeared around 38 kya^5^, although there is a possible indication of their persistence in the Urals until 31-34 kya^66^.

Despite the simplifications and approximations adopted in our model, the main result we obtain of an almost complete genetic substitution within the reasonable 10,000-30,000 years coexistence range as a consequence of recursive small-scale *H. sapiens* immigrations, is reliable and may contribute to explain the Neanderthal disappearance. Interestingly, by using extant hunter-gatherers data^61^ on the mean time between successive live births per woman (4-8 years) and the survival rate (about 0.5) to age 15 (i.e., when entering into the reproductive subpopulation), we can estimate the exit mean lifetime to be $\tau _E \approx 24$ years (as follows from $\dot{N} = 0$). This value is in line with the necessary condition $\Delta t/\tau _E > 2$ (see Subsection "The effect of *Homo sapiens* immigration cycles on the equilibrium genetic subgroup distribution") when using $\Delta t \ge 50$ years as in Fig. 5. Finally, it should be noted that when *H. sapiens* homozygote immigration cycles are terminated (that is, for $t > T_f$), the genetic subgroup distribution of the expected population becomes fully stable (i.e., stationary), thus possibly preserving traces of Neanderthal ancestry in all future generations. This condition, corresponding to a largely homogeneous genetic composition among populations exchanging individuals, can explain the small amount of Neanderthal DNA in the present Eurasian human population.

It is worth to remark that our model has some affinity with the classical continent-island framework in population genetics^45–48^. Indeed, both models share the general feature of unidirectional gene flow from a large source into a smaller recipient population, and both predict convergence of the recipient’s genetic composition toward that of the source. However, if the general convergence behavior resembles the classical continent-island dynamics (Eqs. 38-40 can be used to obtain the allele distribution within the expected population as a function of the immigration cycles, resulting fully equivalent to the classical continent-island expression), our model introducing the concept of the reference tribes ensemble (ideally representing the Neanderthal metapopulation) provided an explicit time-dependent and demographically grounded description of the genotype distribution within the expected population that we consider representative of the dynamics and genetics of the Neanderthal metapopulation. Moreover, the model allowed us to investigate the effects of the statistical noise on a single reference tribe as well as on the mean population of a finite size ensemble, thus offering new quantitative predictions for the pace and extent of genetic replacement under neutral gene flow with parameters directly related to prehistoric human population dynamics.

The fascinating scenario provided by the mathematical model, showing that Neanderthal disappearance rather than a true extinction might be conceived as the result of genetic dilution due to the assimilation with a demographically much larger species, cannot be taken as a possible exclusive explanation. In fact, it is likely that other processes (such as those invoked by the demographic and competition hypotheses) may have played a role to varying extents in different regions and periods. For example, although there is evidence supporting the action of negative selection on Neanderthals^3^, we adopted a neutral model to isolate the demographic and genetic effects of recurrent gene flow alone. This allowed us to establish a conservative baseline: if genetic replacement can occur without selection, then any selective pressure would only enhance the efficiency of this process. Therefore, our neutral drift model can be viewed as a lower-bound scenario, showing that genetic dilution could occur even in the absence of any selective pressure. In summary, the presented model and its implications simply show that genetic admixture can provide another robust explanation for the observed Neanderthal demise, but does not exclude that other factors may have played a substantial role in the disappearance of Neanderthals.

Finally, we would like to note that the genetic dilution model we presented in this paper may be adapted to describe the effects of hybridization in other species including imperiled ones^67^.

## Conclusion

The mathematical model we presented provides a plausible explanation for the gradual disappearance of Neanderthals, suggesting that their genetic assimilation with *H. sapiens*, a demographically much larger species, may have been a significant factor in their decline. Rather than sudden extinction, our model proposes that repeated cycles of *H. sapiens* immigration leading to the Neanderthal gene dilution, could account for the Neanderthals’ disappearance and the observed patterns of Neanderthal ancestry in modern human populations. Although this model provides a possible robust genetic explanation, it is important to note that it does not exclude other possible contributing factors that were not considered in our approach, such as environmental changes, competition, or demographic fluctuations. Evidence of interbreeding and genetic introgression supports the notion that *H. sapiens* and Neanderthals interacted extensively over thousands of years. Therefore, the interplay of genetic, demographic, and environmental factors likely contributed to the complex process of Neanderthal demise.

Future studies incorporating both genetic and archaeological data will be crucial in refining our understanding of this pivotal moment in human evolution.

## Supplementary Information

Supplementary Information.

## Data Availability

The datasets used and/or analyzed during the current study are available from the corresponding author A.A. on reasonable request.
