# Supplementary material for: A simple analytical model for Neanderthal disappearance due to genetic dilution by recurrent small-scale immigrations of modern humans
Source: Sci Rep. 2025 Nov 4;15:38593. doi: 10.1038/s41598-025-22376-6 (PMC12586505; doi:10.1038/s41598-025-22376-6)
Supplement: Supplementary file 1 — Supplementary Information. [file 41598_2025_22376_MOESM1_ESM.pdf]

Appendix

In this appendix, we show how the logistic growth equation can be obtained from the population dynamics equation used in our paper (Eq. 1 of the article) by expressing the rate constatnts k_E and γ as linear functions of N.

When considering k_E and γ as functions of the expected population, we can use for both the Taylor expansion around N_{eq} truncated to the first order terms to obtain

$$k_E = k_E^0 + \eta(N - N_{eq}) \quad \text{(A-1)}$$

$$\gamma = \gamma^0 + \beta(N - N_{eq}) \quad \text{(A-2)}$$

with k_E^0, γ^0 the rate constant values at $N = N_{eq}$ and $\eta, \beta \geq 0$ the corresponding first derivatives, providing

$$\dot{N} = \frac{k}{4} N^2 - \Big[k_E^0 + \eta(N - N_{eq})\Big] N - \Big[\gamma^0 + \beta(N - N_{eq})\Big] (N - N_{eq})$$

$$= -\Big[\eta + \beta - \frac{k}{4}\Big] N^2 + \Big[N_{eq}\eta + 2N_{eq}\beta - k_E^0 - \gamma^0\Big] N + N_{eq}(\gamma^0 - N_{eq}\beta) \quad \text{(A-3)}$$

By assuming $N_{eq}(\gamma^0 - N_{eq}\beta) = 0$ and hence $\gamma^0 = N_{eq}\beta$, we can retrieve the logistic equation

$$\begin{aligned} \dot{N} &= -\Big[\eta + \beta - \frac{k}{4}\Big] N^2 + \Big[N_{eq}(\eta + \beta) - k_E^0\Big] N \\ &= N\Big[N_{eq}(\eta + \beta) - k_E^0\Big] \Big[1 - \frac{(\eta + \beta - k/4)N}{N_{eq}(\eta + \beta) - k_E^0}\Big] \\ &= Nr\Big(1 - \frac{N}{K_C}\Big) \quad \text{(A-4)} \end{aligned}$$

where

$$r = N_{eq}(\eta + \beta) - k_E^0 \quad \text{(A-5)}$$

$$K_C = \frac{N_{eq}(\eta + \beta) - k_E^0}{\eta + \beta - k/4} \quad \text{(A-6)}$$

with $r, K_C > 0$ and thus $\eta + \beta > k/4,\ N_{eq}(\eta + \beta) > k_E^0$.

Once realizing that from Eq. A-3 $N_{eq} = 4k_E^0/k$, we readily obtain $K_C = 4k_E^0/k$ showing that the carrying capacity K_C corresponds to the equilibrium population N_{eq}. It should be noted that the condition $\gamma^0 = N_{eq}\beta$ (necessary to retrieve the logistic equation) implies that for $\beta = 0$ no migration term is present.
